# The way we remember and report: an experiment testing cultural differences in eyewitness memory

**DOI:** 10.1080/09658211.2025.2505213

**Published:** 2025-05-15

**Authors:** Gabi de Bruïne, Annelies Vredeveldt, Peter J. van Koppen

**Affiliations:** Faculty of Law, Vrije Universiteit Amsterdam, Amsterdam, The Netherlands

**Keywords:** Cross-cultural differences, eyewitness memory, high vs. low context communication, acquiescence response style

## Abstract

More and more people report their memories in cross-cultural contexts, including eyewitness interviews. In a pre-registered experiment (*N* = 64), we examined cultural differences in mock eyewitness reports, comparing Sub-Saharan African participants to a matched Western European group. Participants were interviewed about a mock crime video. We assessed differences in the number of correct, incorrect, subjective and total details, the type of details (person, action, object, surrounding), and accuracy. European participants provided significantly more details than African participants. Surprisingly, in free recall African participants used non-significantly more words to provide non-significantly fewer details. An exploratory analysis revealed that this may be due to the fact that Africans included more information that is not directly relevant to the event, such as moral evaluations. That finding supports existing literature on cultural differences in high- versus low-context communication styles. We found no significant differences between groups in the accuracy of witness reports. Because factual details about the event are typically required for criminal investigations, future research should assess how to elicit those from African individuals. Our findings emphasise the importance of considering cultural differences in memory reports and provide insight into the mechanisms underlying such cultural differences.

Cultural differences in memory reports and communication styles can pose challenges for legal decision-makers in cross-cultural settings (Combs, 2010, 2017; De Bruïne et al., 2023; Hope et al., 2022). Around the world, witness statements are often crucial in investigations and legal decision-making processes, such as asylum applications and international criminal cases. If a statement does not align with the assessor's expectations, it may lead to incorrect evaluations, which can have serious consequences (e.g., an unjustified rejection of an asylum claim, or an unwarranted acquittal in a criminal case).

Eyewitness memory in cross-cultural contexts has recently become a topic of interest in the field of legal psychology (see e.g., Anakwah et al., 2020b, 2024; Hope et al., 2023) but experimental evidence on cultural differences in eyewitness memory is still scarce. Prior to the growing interest in cross-cultural research among legal psychologists, there was already a great deal of research on the influence of culture on memory (for an overview, see Wang, 2021). Notable cultural differences highlighted in the literature are the length, specificity, focus, and emotionality of memory reports. Individuals from more individualistic societies (e.g., USA and Europe) tend to provide more detailed, emotionally expressive accounts that focus on themselves. In contrast, individuals from more collectivistic societies (e.g., Asia and Africa) tend to provide less detailed, emotionally neutral accounts that focus on social and contextual aspects (Anakwah et al., 2024, 2020b; Jobson & O'Kearney, 2008; Wang, 2001).

Hofstede’s (1980) framework of cultural dimensions describes systematic differences in perception, attitudes, and behaviour between people from different countries. The most relevant dimensions for investigative interviewing in cross-cultural settings are individualism-collectivism and power distance, as these dimensions describe how individuals relate to and interact with other people and authorities. The individualism-collectivism dimension refers to the degree to which individuals feel independent (i.e., individualism) or interdependent (i.e., collectivism) as part of larger social groups in society, reflecting in valuing more the autonomous self (“I”; individualism) or group cohesion (“we”; collectivism; Hofstede, 2001; Triandis, 2001). The power distance dimension refers to the extent to which individuals acknowledge and anticipate an unequal distribution of power. In societies with high power distance, there is an expectation and acceptance of a distinct hierarchical structure. In contrast, in societies with low power distance, there is an expectation of equality, and differences in power must be justifiable (Hofstede, 2001). In addition to these cultural dimensions, the underlying concepts often used to explain cultural differences in memory reports include cognitive style (Witkin & Berry, 1975), perceptual processing (Masuda & Nisbett, 2001), high- vs. low-context communication style (Hall, 1976), and acquiescence response style (Cheung & Rensvold, 2000). These will be explained in turn below.

There appears to be a dichotomy in these concepts. Some are related to how we perceive, pay attention to, and remember specific details, while others are related to communication style and reporting tendencies. Before providing a statement, an individual has witnessed an event. Previous studies have shown that there are cultural differences in how people perceive a scene and on which elements of the scene they focus. The widely known distinction between cognitive styles is field-independent and field-dependent (Witkin & Berry, 1975). People from individualistic cultures tend to have a field-independent cognitive style, by focusing on specific elements in a scene. In contrast, people from collectivistic cultures tend to have a field-dependent cognitive style, by focusing more on the environment and context. In line with these concepts, research indicates that individuals’ cultural orientation can influence their perceptual processing and the content of their reports (Boduroglu et al., 2009; Istomin et al., 2014; Masuda & Nisbett, 2001). For example, Japanese participants (i.e., collectivistic) tend to process information in a more holistic manner, by focusing on the background and relationships within a scene, whereas American participants (i.e., individualistic) tend to process information more analytically, by focusing on individual elements within the scene (Masuda & Nisbett, 2001). Findings from a recent cross-cultural comparison between eleven countries did, however, not align with predictions from the perceptual processing and cognitive style theory (Lacko et al., 2025). The authors warrant against the straightforward application of this theory across diverse cultural contexts and suggest re-evaluation of its generalisability (Lacko et al., 2025).

Cross-cultural differences in memory reports could also appear due to preferences in reporting tendencies and communication styles. People from collectivistic cultures tend to have a more interdependent self-construal (Markus & Kitayama, 1991), resulting in less detailed memory reports that focus on relationships and social context (Jobson & O'Kearney, 2008; Marian & Kaushanskaya, 2004; Wang, 2001). On the contrary, people from individualistic cultures tend to have a more independent self-construal (Markus & Kitayama, 1991), resulting in detailed memory reports that focus on the self (Jobson & O'Kearney, 2008; Marian & Kaushanskaya, 2004; Wang, 2001). Associated with self-construal is the tendency for self-enhancement or self-eﬀacement in self-presentation (Takata, 2003). People from collectivistic societies are more modest in their self-presentation (i.e., self-effacement), whereas people from individualistic societies are less modest in their self-presentation (i.e., self-enhancement; Heine et al., 2000).

Different communication styles could also account for cultural differences in memory reports. After all, witness statements are taken in an interview and are therefore subject to interaction between people. Hall (1976) proposed two communication styles: high-context communication and low-context communication. In the low-context communication style, typical for individualistic cultures, communication is explicit and direct and focused on providing content. In the high-context communication style, typical for collectivistic cultures, communication is more implicit and focused on providing context (Hall, 1976). Power distance can also influence communication in an interview. Mock witnesses from a low power distance culture (i.e., the Netherlands) provided more details when they thought they were reporting to the police rather than to a peer (Anakwah et al., 2024). In contrast, for mock witnesses from a high power distance culture (i.e., Ghana), no significant differences in the number of details provided in each reporting setting (police versus peer) were observed. Anakwah and colleagues argue that witnesses usually attempt to provide more detailed information to the police during an investigation, to be helpful. However, this tendency may be hindered among Ghanaian witnesses due to the perceived authority of the interviewer, as high power distance can impair free and spontaneous communication (Anakwah et al., 2024; Ghosh, 2011; Vredeveldt et al., 2023).

Another relevant concept in cross-cultural communication is acquiescence response style, which refers to the tendency to agree or response in the affirmative due to social desirability (Cheung & Rensvold, 2000). An acquiescence response style for sub-Saharan African participants was observed in studies on cross-cultural differences in object recognition (De Bruïne et al., 2018) and lineup performance (De Bruïne et al., 2025).

Based on the limited number of cross-cultural studies on eyewitness memory to date, Hope et al. (2023, p. 3) observed a “tendency towards underreporting of details” in memory reports provided by individuals from sub-Saharan African countries in mock crime paradigms (see Anakwah, 2021). In their study, Hope and colleagues also found this pattern of underreporting comparing statements of Lebanese (i.e., collectivistic) and UK (i.e., individualistic) participants. Given that detailed accounts are crucial for investigators to solve crimes, more research is needed to replicate the findings, strengthen the evidence base, and gain more insight into the underlying mechanisms of observed differences in memory reports of different cultural groups.

## Current study

In the current study, we follow Hofstede (2011, p. 3) in defining culture as: “the collective programming of the mind that distinguishes the members of one group or category of people from others”, and it was operationalised as the distinction between the two participant groups in terms of region of origin (i.e., Western Europe or sub-Saharan Africa). We assessed differences in memory reports between participants from sub-Saharan Africa and participants from Western Europe in a mock crime paradigm in which participants witnessed an event on video. Participants were then interviewed to explore what aspects of the event they retrieved and how they reported about the event. The participant groups were matched on four demographic variables: sex, age, level of education, and urban/rural living environment.

The language in which an interview is conducted can influence what people report (Ewens et al., 2016; Marian & Kaushanskaya, 2004). The use of an interpreter tends to reduce the number of details provided in an interview (Ewens et al., 2016). Because an analysis of the role of language was outside the scope of this paper, the language factor was neutralised by conducting all interviews in English. Participant groups were therefore also matched on language proficiency (e.g., a sub-Saharan African participant with intermediate English proficiency was matched to a Western European participant with intermediate English proficiency), making language the fifth matching criterion.

Based on the literature in cross-cultural psychology and on the recent handful of experiments on cross-cultural differences in eyewitness memory, we expected that sub-Saharan African participants would provide less detailed reports (Anakwah et al., 2020b, 2024; Hope et al., 2023; Wang, 2001), due to their collectivistic background associated with a more interdependent self-construal (Markus & Kitayama, 1991). Furthermore, we predicted that sub-Saharan African participants would retrieve more information related to social and contextual aspects of the incident than Western European participants (Jobson & O'Kearney, 2008), due to their more field-interdependent cognitive style, holistic perceptual processing, and high-context communication style (Hall, 1976; Masuda & Nisbett, 2001; Witkin & Berry, 1975). Since the literature provides no solid basis for making predictions about cultural differences in the accuracy of eyewitness reports, we formulated no specific hypotheses for accuracy.^1^

## Method

This research was preregistered via the Open Science Framework (OSF), available at https://osf.io/uxnfq/.^2^ The data are available at https://osf.io/ehs93.

### Inclusion criteria

Participants were allowed to take part in the study if they were at least eighteen years old and had sufficient English proficiency to understand the research and participate in an interview. We used the same categorisation as Ewens et al. (2016) to rate participants’ English proficiency. The scale ranged from 1 (i.e., beginner; those who know a few English words) to 5 (i.e., upper-intermediate; those who can talk fluently and almost completely accurately). In order to participate, participants needed a minimal level of 3 (i.e., pre-intermediate; those with a good basic ability to communicate and understand many subjects). Unlike Ewens and colleagues, we decided to have the interviewers assess English proficiency rather than participants themselves, to avoid any potential cultural differences in self-evaluations. Because the data were collected in Western Europe, there was an additional inclusion criterion for sub-Saharan African participants, namely that they had lived for a maximum of two years in Western Europe, to reduce potential effects of acculturation (Anakwah et al., 2020a).

### Participants

We conducted an a priori power analysis. Wang (2001) examined several dependent variables relevant to our variables of interest, including the number of details reported (i.e., number of words and number of propositions) in a study on cultural differences in autobiographical memory reports. Cohen’s *d* effect sizes for cultural differences on those variables ranged from 0.70 to 1.37, with an average *d* of 1.01. To detect an effect of the same size as the smallest of those effect sizes (*d* = 0.70) with power = .80, we needed to recruit 32 participants per condition. As a result of the matching procedure, we had an equal distribution in the two groups in terms of sex and rural/urban living area. In addition, there were no significant differences between the groups in terms of participant age, *U* = 500.50, *p* = .877, *η*^2^ = .0004; education level, χ^2^(2) = .99, *p* = .608, Cramer's *V* = .125; or English proficiency, χ^2^(2) = 1.35, *p* = .510, Cramer's *V* = .145.

Thirty-two sub-Saharan African participants (24 male and 8 female) were recruited through educational institutions and churches in the Netherlands. They had come from Ghana (*n* = 14), Cameroon (*n* = 4), Democratic Republic of Congo (*n* = 4), Nigeria (*n* = 4), Ethiopia (*n* = 2), Kenya (*n* = 1), Malawi (*n* = 1), Tanzania (*n* = 1), and Uganda (*n* = 1). Most of them indicated that they originated from a city (*n* = 27) and a few from a village (*n* = 5). The sub-Saharan African participants in our sample had been in Western Europe for 8.22 months on average (*SD* = 6.91; range 0–24 months). They were between 18 and 49 years old (*M* = 29.84; *SD* = 7.71). The highest level of education each of them had completed varied from high school (*n* = 4), secondary vocational education (*n* = 1), to higher vocational education and university (*n* = 27). Their English proficiency ranged from category 3 (pre-intermediate) to 5 (upper-intermediate; *M* = 4.41; *SD* = 0.67).

Western European participants were selected based on their match to the sub-Saharan African participants in our sample in terms of sex, age, education level, rural/urban living area, and English proficiency. Thirty-two Western European participants (24 male and 8 female) were recruited via the researchers’ personal networks at different locations in the Netherlands. They had come from the Netherlands (*n* = 28), Germany (*n* = 3), and France (*n* = 1). As a result of the matching, 27 of them originated from a city and five from a village. Western European participants’ ages ranged from 19 to 59 (*M* = 30.09; *SD* = 9.32). The highest level of education they had completed varied from high school (*n* = 7), secondary vocational education (*n* = 1), to higher vocational education and university (*n* = 24). Their English proficiency ranged from category 3 (pre-intermediate) to 5 (upper-intermediate) (*M* = 4.41; *SD* = 0.76).

### Materials

#### Mock crime video

We showed participants a 70-second video created specifically for the present study. The video depicted a theft in a park: two individuals steal a woman's backpack while she is assisting an injured runner. One of the perpetrators is a black man from Gambia and the other is a white man from the Netherlands. The video was presented without sound.

In a cross-cultural mock crime experiment involving participants from Ghana and the Netherlands, participants performed better when recalling crimes witnessed in their native setting compared to those seen in a non-native setting (Anakwah et al., 2020b). In order to mitigate this effect, we decided to record the video outside, without sound, and with actors from a sub-Saharan African and a Western European country, to create a stimulus that was as culturally neutral as possible.

#### Cultural Orientation Scale

To assess the self-reported cultural orientations of participants, we used the Cultural Orientation Scale (Triandis & Gelfand, 1998). Triandis and Gelfand argue that both individualism and collectivism can be divided into two types: horizontal (emphasising equality) and vertical (emphasising hierarchy). This results in four subscales of the test: Horizontal Individualism, Vertical Individualism, Horizontal Collectivism, and Vertical Collectivism. In Horizontal Individualism, people seek to differentiate themselves from groups, value independence, but are not focused on achieving high status (e.g., “I'd rather depend on myself than others”). In Vertical Individualism, people often strive to differentiate themselves and achieve status through competition (e.g., “It is important that I do my job better than others”). In Horizontal Collectivism, people perceive themselves as similar to others and emphasise common goals, but they are not readily submissive to authority (e.g., “If a coworker gets a prize, I would feel proud”). In Vertical Collectivism, people prioritise their group's well-being and tend to comply with orders from authorities (e.g., “Parents and children must stay together as much as possible”).

All four subscales have four statements (i.e., 16 items in total) in the test. Participants were instructed to indicate the extent to which each statement was true about themselves or the view they uphold and that their responses should range between 1 (“never or definitely no”) to 9 (“always or definitely yes”).

### Procedure

Participants took part individually and signed an informed consent form prior to watching the mock crime video. As part of the informed consent, participants were informed that the researcher would show them a video of an incident. They were instructed to watch the video carefully and informed that there would be no sound. After a brief filler task (i.e., Spot the Differences), participants were interviewed. All participants were instructed to provide as much information as possible in as much detail as possible. The interview started with a free recall (i.e., “Please tell me what happened in the video”), followed by some open-ended follow-up questions focusing on aspects already mentioned in the free recall (cued-recall phase). The cued-recall questions started general (e.g., “You told me about a woman. Please tell me more about that woman”) and became more specific (e.g., “What was she wearing?”). The cued-recall questions related to the following categories: persons (i.e., appearances, actions, relationships, interactions), objects and surroundings. The interview ended with the question whether participants wanted to add something to their statements. Next, participants performed two person-identification lineups and two object-identification lineups as part of a larger data collection effort for an eyewitness identification project, which will be addressed in a separate article. After the lineup tasks, participants completed the Cultural Orientation Scale to measure their self-reported individualism and collectivism (Triandis & Gelfand, 1998). Lastly, participants completed a short demographic questionnaire. Upon completion, they were debriefed and offered €20 to compensate their time. The study received ethical approval from the Ethics Committee of Legal and Criminological Research of the Faculty of Law at the Vrije Universiteit Amsterdam.

### Coding

All interviews were audio-recorded and transcribed. We developed an elaborate coding scheme listing as many details from the video as possible. While coding, the main coder progressively added additional details mentioned by the participants that were not in the original coding scheme. Transcripts were coded for accuracy (correct, incorrect, subjective) as well as detail type (person, action, object, surrounding; Vredeveldt et al., 2018). The final coding scheme contained 867 details: 532 person details (e.g., “He had a beard”), 153 action details (e.g., “He was jogging”), 117 object details (e.g., “It was pink”), and 65 surrounding details (e.g., “It was in the woods”). The main coder first coded all transcripts. Once the coding scheme had been finalised, a random sample of 25% of the interviews were coded by a second coder. The main and second coder reached substantial agreement on both accuracy, *κ* = .74, *p* < .001, and detail type, *κ* = .77, *p* < .001. Both coders were blind to Participant Group.

## Results

Before conducting the analyses, we checked all relevant statistical assumptions and transformations were applied where necessary. Square-root transformation of the raw frequencies solved most problems with the assumptions. In case the transformation did not solve non-normality or inequality of variance, we performed nonparametric tests and compared the results to the parametric tests, reported in footnotes in the relevant sections. We report descriptive data for the untransformed variables to facilitate interpretation of the findings. All reported *p* values are two-tailed. The benchmarks for effect size are .01 ≤ *η*^2^ < .06 for a small effect, .06 ≤ *η*^2^ < .14 for a medium effect, and *η*^2^ ≥ .14 for a large effect (Cohen, 1988).

### Number of details

**Figure 1.**
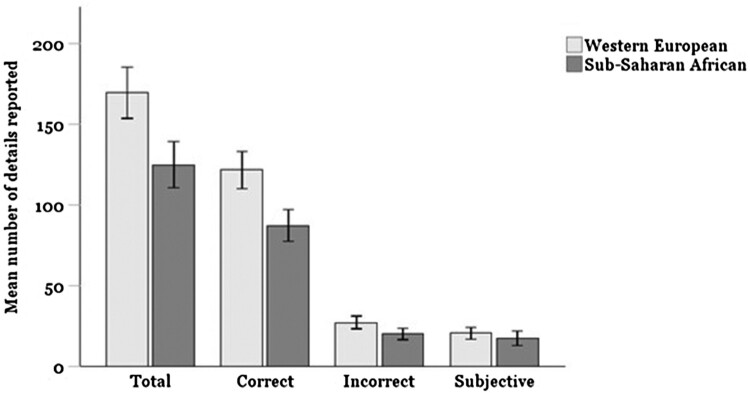
Total number, correct, incorrect, and subjective details per cultural group. Note: Error bars indicate 95% confidence intervals.

A one-way MANOVA was conducted to determine whether there is a difference between sub-Saharan African and Western European participants in the number of correct, incorrect, and subjective details reported (see Figure 1). There was a significant multivariate effect of cultural background on the number of details reported, *V* = 0.29, *F*(3, 60) = 8.15, *p* < .001, *η*^2^ = .289. Western European participants (*M* = 169.59; *SD* = 44.06) reported nearly 45 more details on average than sub-Saharan African participants (*M* = 124.97; *SD* = 29.82). This was reflected in significantly more correct details, *F*(1, 62) = 24.77, *p* < .001, *η*^2^ = .285, as well as significantly more incorrect details, *F*(1, 62) = 8.75, *p* = .004, *η*^2^ = .124. There was no significant difference in the number of subjective details reported, *F*(1, 62) = 2.48, *p* = .121, *η*^2^ = .038.

### Accuracy

In terms of accuracy (i.e., the percentage of details that was correct), a one-way ANOVA showed that Western European participants (*M* = 72.00%; *SD* = 6.21%) and sub-Saharan African participants (*M* = 70.21%; *SD* = 7.82%) did not differ significantly, *F*(1, 62) = 1.03, *p* = .314, *η*^2^ = .016. Thus, Western European participants provided significantly more details than sub-Saharan African participants, but their reports were not significantly more accurate.

### Detail types

We conducted a one-way MANOVA to determine whether there is a difference between the two cultural groups in the number of person, action, object and surrounding details reported. There was a significant multivariate effect of cultural background, *V* = .493, *F*(4, 59) = 14.35, *p* < .001, *η*^2^ = .493. This was reflected in significantly more person details reported by Western European participants (*M* = 89.00; *SD* = 25.54) compared to their sub-Saharan African counterparts (*M* = 58.38; *SD* = 23.11), *F*(1, 62) = 30.69, *p* < .001, *η*^2^ = .331. In addition, Western European participants (*M* = 27.00; *SD* = 9.04) reported significantly more object details than sub-Saharan African participants (*M* = 22.44; *SD* = 8.33), *F*(1, 62) = 4.65, *p* = .035, *η*^2^ = .070. Moreover, Western European participants (*M* = 19.03; *SD* = 7.28) also reported significantly more surrounding details than sub-Saharan African participants (*M* = 10.88; *SD* = 5.58), *F*(1, 62) = 29.16, *p* < .001, *η*^2^ = .320.^3^ Lastly, there was no significant difference between Western European (*M* = 34.44; *SD* = 9.24) and sub-Saharan African participants (*M* = 33.06; *SD* = 10.04) in the number of action details reported, *F*(1, 62) = 0.42, *p* = .522, *η*^2^ = .007.

### Recall phases

We also examined whether distinct patterns were observed in different recall phases. A one-way MANOVA on the effect of cultural background on the number of details in free and cued recall, respectively, revealed a significant multivariate effect, *V* = .239, *F*(2, 61) = 9.55, *p* < .001, *η*^2^ = .239. In the free-recall phase, we observed no significant difference in the number of details, *F*(1, 62) = 1.66, *p* = .203, *η*^2^ = .026. Thus, the multivariate effect was driven by the cued-recall phase, in which Western European participants provided significantly more details (*M* = 119.03; *SD* = 38.95) than sub-Saharan African participants (*M* = 80.06; *SD* = 32.28), *F*(1, 62) = 18.99, *p* < .001, *η*^2^ = .234.

We examined whether this difference could potentially be explained by the number of questions posed to each cultural group in cued recall, but found no significant difference in the number of questions (Western European participants: *M* = 34.88; *SD* = 6.38; sub-Saharan African participants: *M* = 33.38; *SD* = 9.83), *F*(1, 62) = .90, *p* = .346, *η*^2^ = .117.^4^

### Number of words

A one-way MANOVA on the effect of cultural background on the number of words in free and cued recall, respectively, revealed a significant multivariate effect (Western European participants: *M* = 1356.44; *SD* = 433.58; sub-Saharan African participants: *M* = 1161.16; *SD* = 462.91), *V* = .103, *F*(2, 61) = 3.52, *p* = .036, *η*^2^ = .103. In the free-recall phase, there was no significant difference between groups in the number of words used, *F*(1, 62) = 0.17, *p* = .680, *η*^2^ = .003.^5^ In the cued-recall phase, however, Western European participants used significantly more words (*M* = 1,127.13; *SD* = 353.96) than sub-Saharan African participants (*M* = 917.94; *SD* = 395.95), *F*(1, 62) = 4.97, *p* = .030, *η*^2^ = .074.

Even though there were no significant differences between groups in the number of words or details used in free recall, the data pattern for these two variables goes in opposite directions. Remarkably, sub-Saharan African participants used non-significantly *more* words (*M* = 243.22; *SD* = 138.22) than Western European participants (*M* = 229.31; *SD* = 130.31) to provide non-significantly *fewer* details (African: *M* = 44.94; *SD* = 16.02; European: *M* = 50.53; *SD* = 18.63). The next section explores potential mechanisms behind this unexpected data pattern.

### Inferences and meta-observations

The pattern described in the previous section raised the question of what the sub-Saharan African participants used their words for, if not to provide codable details. We therefore conducted an exploratory analysis of the information provided by participants that had not been coded as correct, incorrect or subjective details. This concerns the type of information that could not be coded based on the main coding scheme, because it did not constitute factual details about the event but rather reflected meta-observations or inferences participants made after watching the video. More specifically, we categorised this type of contextual information into the following categories: (1) Scam; (2) Speculation; (3) Guessing; (4) Not knowing/not remembering; (5) Uncertainty/not sure; (6) Certainty/confidence; and (7) Moral lesson (see Table 1). For two of these variables, sub-Saharan Africans scored significantly higher than Western Europeans: Scam and Moral lesson. For two other variables, Western Europeans scored significantly higher than sub-Saharan Africans: Guessing and Uncertainty. These four variables will be discussed in further detail below.

**Table 1.** Average number of references to different types of contextual information per interview, by cultural group.

Context	Western European		Sub-Saharan African		Difference between groups
	*M*	*SD*	*M*	*SD*	
Scam	2,00	2,33	4,16	4,68	*F*(1, 62) = 4.30, *p* = .042, *η*^2^ = .065
Moral lesson	0,00	0,00	0,88	1,56	*F*(1, 62) = 15.69, *p* < .001, *η*^2^ = .202
Speculation	3,75	4,24	4,94	5,66	*F* < 1
Not knowing	7,75	4,62	7,56	4,62	*F* < 1
Guessing	7,94	4,61	4,94	4,30	*F*(1, 62) = 8.45, *p* = .005, *η*^2^ = .120
Uncertainty	23,31	14,26	14,59	11,43	*F*(1, 62) = 9.10, *p* = .004, *η*^2^ = .128
Certainty	2,53	2,95	3,28	3,92	*F* < 1

#### Scam

We coded the provided information as “Scam” if the participant indicated that the theft was a scam, for example by stating that the injured runner was part of the perpetrator group. As shown in Table 1, sub-Saharan African participants were significantly more likely to provide details about a scam than Western European participants.^6^ The following two quotes from sub-Saharan African participants illustrate the often lengthy way in which they reported the alleged scam:

It was a kind of arrangement between him and the other two guys. The three guys, I believe … It looks like, it looks like, uhm, it looks like it was a planned thing … Okay. It looks like it was a planned thing. But you can, you can see that, nothing happened to him, and all of a sudden he just squatted. So it looks like the three guys were standing somewhere, they planned that the first guy would go and pretend as if something has hurt him and then, uhm, would take the girls attention, you considered that relationship, as that just between the three people. And, uhm, the two guys communicated, and then with the third guy. So it’s a planned thing.^7^

Now, she must have a friend who released information about her, because, they studied her weakness, to understand that okay, she should be a kind person, who would pay attention to somebody who was hurt, and the other thing is that, there was a girl in the scene, in the group, then they used the man, they used the power of the opposite sex, to win her attention. So either, she has two weaknesses. Either, she’s very attracted to men, or she’s very kind person. So they used that as a weakness to distract her.^8^

#### Moral lessons

We coded information as a moral lesson if the participant provided a moral evaluation of the situation. There was a significant difference between sub-Saharan African and Western European participants in the number of moral lessons expressed (see Table 1).^9^ Western European participants did not provide any moral evaluations, reflecting a floor effect, whereas sub-Saharan African participants did provide some moral evaluations in their reports. To illustrate the way moral evaluations were reported, we provide two quotes from different transcripts:

[…] you should be cautious, if you want to help somebody, especially in isolated places, you have to be vigilant, it’s the lesson I draw from this video. Because at times you want to help somebody, when the person is actually exposing you to danger. So you have to be attentive, with your generosity.^10^

[…] is all about I could say it’s trying to teach, it’s teaching in some way that you should be vigilant, irrespective of how much you want to help people. Don’t forget your own stuffs behind in helping people because not everyone has the same nature of how act like you might have […]^11^

#### Guessing and uncertainty

We coded the provided information as “Guessing” if the participant provided an indication that the answer was a guess (e.g., “I guess the can was pink”) and as “Uncertainty” if the participant indicated that they were not sure about their answer (e.g., “It could have been a scarf, but I am not sure”). Western European participants were significantly more likely to indicate they were guessing or uncertain than sub-Saharan African participants (see Table 1). Potential explanations for this finding will be addressed in the Discussion.

#### Cultural Orientation Scale

To assess self-reported cultural orientation, participants completed the Cultural Orientation Scale. We conducted a one-way MANOVA to determine the effect of cultural background on the four subscales of the Cultural Orientation Scale. There was a significant multivariate effect on cultural background, *V* = .243, *F*(4, 59) = 4.73, *p* = .002, *η*^2^ = .243. This manifested itself in a significant difference on the Horizontal Individualism subscale, *F*(1, 62) = 6.23, *p* = .015, *η*^2^ = .091.^12^ Surprisingly, sub-Saharan African participants (*M* = 27.69; *SD* = 6.00) scored significantly higher on Horizontal Individualism than Western European participants (*M* = 24.13; *SD*  = 4.26), indicating that sub-Saharan African participants rated those statements (e.g., “I'd rather depend on myself than others”) higher on average than Western European participants. Sub-Saharan African participants (*M* = 28.69; *SD* = 7.12) also scored significantly higher on Vertical Collectivism than Western European participants (*M* = 22.38; *SD* = 5.32), indicating that sub-Saharan African participants rated those statements (e.g., “Parents and children must stay together as much as possible”) on average higher than Western European participants, *F*(1, 62) = 13.44, *p* < .001, *η*^2^ = .178.^13^ We found no significant difference between the cultural groups on the Vertical Individualism subscale, *F*(1, 62) = 0.44, *p* = .509, *η*^2^ = .007, and the Horizontal Collectivism subscale *F*(1, 62) = 1.70, *p* = .197, *η*^2^ = .027.^14^ Overall, sub-Saharan African participants (*M* = 104.72; *SD* = 15.51) rated the statements across the four subscales significantly higher than Western European participants (*M* = 91.75; *SD* = 12.00), *F*(1, 62) = 14.00, *p* < .001, *η*^2^ = .184.

We assessed whether there was a correlation between the scores on the Cultural Orientation Scale and the number of details provided and number of words used. The total score on the Cultural Orientation Scale was found to be moderately negatively correlated to the number of details provided, *r*(62) = −.341, *p* = .006, as well as weakly negatively correlated to the number of words used, *r*(62) = −.255, *p* = .042. The higher the score on the Cultural Orientation Scale, the fewer details and words were provided. This aligns with the previously noted differences in the detail quantity and word count between sub-Saharan African and Western European participants.

## Discussion

We assessed differences in memory reports between sub-Saharan African and Western European participants in a mock crime paradigm. Our results revealed four main findings. First, Western European participants reported significantly more details than sub-Saharan African participants, but we found no significant differences in accuracy. Thus, Western European participants reported more correct, but also more incorrect details than sub-Saharan African participants. Second, while Western Europeans provided more details and used more words than sub-Saharan Africans in the cued-recall phase, we found no significant differences in the number of details or words in the free-recall phase. Exploratory analyses revealed that sub-Saharan African participants tended to use their words to report more contextual information instead of codable information about the core of the event. Third, Western European participants provided significantly more person, object, and surrounding details, but not more action details than sub-Saharan African participants. Fourth, sub-Saharan African participants scored significantly higher on the cultural orientation scale (across all subscales) than Western European participants. Each finding will be discussed in turn.

Our finding that Western European mock witnesses provided more details than sub-Saharan African mock witnesses replicates previous findings with sub-Saharan African mock witnesses (Anakwah, 2021) and mock witnesses from other collectivistic societies (see e.g., Lebanese vs. British: Hope et al., 2023; Chinese vs. Israeli-Arab vs. British: Leal et al., 2018; Arab vs. British: Vrij et al., 2020). However, Western European participants did not necessarily use significantly more words to provide more details. Closer examination of our data revealed distinct patterns in free and cued recall. While Western Europeans provided significantly more details and used more words than sub-Saharan Africans in the cued-recall phase, we found no significant differences in the number of details or words in the free-recall phase. A potential explanation for the observed difference in cued recall could be that interviewers asked Western European participants more questions, but this explanation was not supported by our data; there was no significant difference in the number of questions posed to each cultural group. This indicates that sub-Saharan African participants responded differently to the questions asked during cued recall, resulting in fewer forensically relevant details in their statements.

Exploratory analyses to investigate this unexpected data pattern showed that sub-Saharan Africans used their words to report more contextual information. This manifested itself in elaborate descriptions of how the whole event was a scam (e.g., the runner was associated with the thieves) and the provision of moral lessons (e.g., how one should never leave their things unattended). These findings support the concept of high- vs. low-context communication (Hall, 1976). Western Europeans, who typically have a low-context communication style, provided more codable information about the event (i.e., content), whereas sub-Saharan Africans, who typically have a high-context communication style, used more words to describe the context around the event, including the evaluative context. Because factual details about the event are crucial for police investigations, future researchers should examine how to elicit more factual details from participants with collectivistic backgrounds. In one promising study, the use of directive versus instructive prompts in multicultural investigative contexts was investigated Kontogianni et al. (2023). Initial results indicate that instructive prompts (i.e., prompts that establish the level of detail required in a response and why) increase the number of correct details provided in a mock crime paradigm compared to directive prompts (i.e., open-ended prompts). The use of instructive prompts should be tested further in different cultural contexts.

Our hypothesis that sub-Saharan Africans would report relatively more information about social and contextual aspects of the incident than Western Europeans (Jobson & O'Kearney, 2008) was only partially supported. Consistent with this hypothesis, we found that Western European participants provided more person and object details than sub-Saharan African participants. That is in line with the more analytical perceptual processing style, focusing on individual elements in a scene, that is usually found in individualistic cultures (Masuda & Nisbett, 2001; Witkin & Berry, 1975). To an extent, our finding that Western Europeans did not report significantly more action details than sub-Saharan Africans could also be interpreted as consistent with the hypothesis, since action details are arguably most related to social relations in the scene. However, our finding that Western Europeans reported more surrounding details than sub-Saharan Africans seems to contradict our hypothesis; after all, details about the surrounding of the incident would typically be considered contextual information. Yet, the exploratory analysis opened our eyes to our own narrow definition of context. Perhaps contextual information is not so much about the surrounding details (which could still be considered part and parcel of the event), but more about the wider context, such as the plans and motives of the people involved and the lessons learned from watching the event. Our results suggest that sub-Saharan Africans may have different cultural expectations and norms concerning the extent to which that wider, evaluative context should be described.

Participants completed the Cultural Orientation Scale to evaluate their self-reported cultural orientation. Sub-Saharan Africans scored significantly higher on two of the four subscales, including, surprisingly, horizontal individualism. Even though this finding is at odds with the expectation that Western Europeans are more individualistic than sub-Saharan Africans, it is in line with previous findings (see e.g., Anakwah, 2021; Hope et al., 2023) and provides support for previously voiced criticisms of using nation-level dimensions to explain individual behaviour (for an overview, see Sharma, 2010). There could also be an alternative explanation. Upon closer inspection, we noticed that sub-Saharan African participants scored significantly higher on all subscales than Western European participants. That could indicate a tendency to agree (i.e., acquiescence response style; Cheung & Rensvold, 2000) with the statements rather than an actual difference in the evaluation of the statements. Another finding from the current study also points towards an acquiescence response style in sub-Saharan African participants. The finding that sub-Saharan Africans were significantly less likely than Western Europeans to say that an answer was a guess or that they were uncertain about it, indicates an acquiescence bias, favouring agreement and harmony in conversations over giving an honest response. Future cross-cultural research should not only consider the validity of self-reported cultural orientation scales but also factor in the potential influence of acquiescence response style among participants from collectivistic cultures.

One limitation of the current study is that the sub-Saharan African participants were recruited and interviewed in the Netherlands. Although we did find differences between their memory reports and those of their Western European counterparts, based on previous findings (Anakwah et al., 2020b) we would expect the differences to be even be more pronounced in a sample of sub-Saharan Africans living in their home country, especially those living in more rural areas. People living in rural areas tend to be more collectivistic than those living in urban areas (Rooks et al., 2016). Another limitation is that all participants were interviewed in English (a non-native language for all), which may have impaired their ability to provide complete reports (Ewens et al., 2016; Marian & Kaushanskaya, 2004). Although the influence of language was beyond the scope of our study, we matched the two cultural groups on language ability to avoid potential confounds with regards to language. Lastly, although we aimed to use a culturally neutral stimulus by using a culturally neutral setting (a park) with actors from both cultural groups (sub-Saharan African and Western European) and presented the video without sound, the video was recorded in the Netherlands. Therefore, some features of the video may have been less familiar to the sub-Saharan African participants than to the Western European participants.

In conclusion, fact-finding in legal cases increasingly depends on communication between people with different cultural backgrounds. Research into cultural differences in memory statements in the context of investigative interviewing is in its early stages, but it is clear from current and previous findings that witnesses from collectivistic societies provide less detailed reports than witnesses from individualistic societies. The current study provides more insight into the mechanisms underlying cultural differences by looking beyond the type of details that are usually coded in research studies. As such, it contributes to the search for a solution for closing the reporting gap between people from different cultural backgrounds.

## Notes

1 In our preregistration, we listed additional hypotheses (3, 4 and 5) related to lineup tasks, which will be assessed in a separate article.

2 Contrary to what we stated in the preregistration, we decided not to code for emotionality in this study. Upon further examination, we found that the stimulus was not suitable for this purpose. We decided to examine this in a separate experiment.

3 Square-root transformation did not solve non-normality for Surrounding details, but a nonparametric test confirmed the parametric finding.

4 Square-root transformation did not solve inequality of variance for Number of Questions in Cued Recall, but a nonparametric test confirmed the parametric finding.

5 Square-root transformation did not solve non-normality for Number of Words Free Recall, but a nonparametric test confirmed the parametric finding.

6 Square-root transformation did not solve non-normality of Scam, but a nonparametric test confirmed the parametric finding.

7 Transcript of participant 33.

8 Transcript of participant 29.

9 Square-root transformation did not solve non-normality or heterogeneity of variances for Moral lesson, but a nonparametric test confirmed the parametric finding.

10 Transcript of participant 9.

11 Transcript of participant 28.

12 Square-root transformation did not solve non-normality for Horizontal Individualism, but a nonparametric test confirmed the parametric finding.

13 Square-root transformation did not solve non-normality for Vertical Collectivism, but a nonparametric test confirmed the parametric finding.

14 Square-root transformation did not solve non-normality for Horizontal Collectivism. We performed a nonparametric test that did not confirm the finding of the parametric test: a Mann-Whitney test showed that Sub-Saharan African participants (*M* = 30.31; *SD* = 5.56; *Mdn* = 28.00) scored significantly higher on the Horizontal Collectivism subscale than Western European participants (*M* = 28.50; *SD* = 3.81; *Mdn* = 29.00), *U* = 346.00, *z* = −2.24, *p* = .025, *r* = −.28.
